# Oropharyngeal microbial ecosystem perturbations influence the risk for acute respiratory infections in common variable immunodeficiency

**DOI:** 10.3389/fimmu.2024.1371118

**Published:** 2024-05-30

**Authors:** Federica Pulvirenti, Maria Giufrè, Tancredi M. Pentimalli, Romina Camilli, Cinzia Milito, Annalisa Villa, Eleonora Sculco, Marina Cerquetti, Annalisa Pantosti, Isabella Quinti

**Affiliations:** ^1^ Reference Center for Primary Immune Deficiencies, Azienda Ospedaliero Universitaria (AOU) Policlinico Umberto I, Rome, Italy; ^2^ Department of Infectious Diseases, Istituto Superiore di Sanità, Rome, Italy; ^3^ Laboratory for Systems Biology of Gene Regulatory Elements, Berlin Institute for Medical Systems Biology (BIMSB), Max Delbrück Center for Molecular Medicine in the Helmholtz Association (MDC), Berlin, Germany; ^4^ Charité—Universitätsmedizin Berlin, corporate member of Freie Universität Berlin and Humboldt-Universität zu Berlin, Berlin School of Integrative Oncology (BSIO), Berlin, Germany; ^5^ Department of Molecular Medicine, Sapienza University of Rome, Rome, Italy

**Keywords:** common variable immunodeficiency, IgA, IgM, microbiome and dysbiosis, Haemophilus, Pneumococcus, chronic obstructive pulmonary disease, oropharyngeal microbiome

## Abstract

**Background:**

The respiratory tract microbiome is essential for human health and well-being and is determined by genetic, lifestyle, and environmental factors. Patients with Common Variable Immunodeficiency (CVID) suffer from respiratory and intestinal tract infections, leading to chronic diseases and increased mortality rates. While CVID patients’ gut microbiota have been analyzed, data on the respiratory microbiome ecosystem are limited.

**Objective:**

This study aims to analyze the bacterial composition of the oropharynx of adults with CVID and its link with clinical and immunological features and risk for respiratory acute infections.

**Methods:**

Oropharyngeal samples from 72 CVID adults and 26 controls were collected in a 12-month prospective study. The samples were analyzed by metagenomic bacterial 16S ribosomal RNA sequencing and processed using the Quantitative Insights Into Microbial Ecology (QIME) pipeline. Differentially abundant species were identified and used to build a dysbiosis index. A machine learning model trained on microbial abundance data was used to test the power of microbiome alterations to distinguish between healthy individuals and CVID patients.

**Results:**

Compared to controls, the oropharyngeal microbiome of CVID patients showed lower alpha- and beta-diversity, with a relatively increased abundance of the order *Lactobacillales*, including the family *Streptococcaceae*. Intra-CVID analysis identified age >45 years, COPD, lack of IgA, and low residual IgM as associated with a reduced alpha diversity. Expansion of *Haemophilus* and *Streptococcus* genera was observed in patients with undetectable IgA and COPD, independent from recent antibiotic use. Patients receiving azithromycin as antibiotic prophylaxis had a higher dysbiosis score. Expansion of *Haemophilus* and *Anoxybacillus* was associated with acute respiratory infections within six months.

**Conclusions:**

CVID patients showed a perturbed oropharynx microbiota enriched with potentially pathogenic bacteria and decreased protective species. Low residual levels of IgA/IgM, chronic lung damage, anti antibiotic prophylaxis contributed to respiratory dysbiosis.

## 1 Introduction

Over the years, the implications of human microbiome changes in health and diseases have been increasingly recognized (1). Technological progress in high throughput sequencing led to the recognition of microbiome-host interactions in maintaining a homeostatic environment of the human immune system (2). Moreover, perturbation in the microbiota architecture, called dysbiosis, has been related to various human diseases (3–5). The airway microbiome is a crucial driver of respiratory homeostasis (6) and is associated with susceptibility to infections, hypersensitivity reactions, and immune-mediated diseases (7). Mucosal immunoglobulins exert multiple immune effector functions in regulating microbiome composition (8, 9). Secretory IgA is crucial to engendering robust host-microbial symbiosis, allowing colonization in mucosal niches through the exclusion of exogenous competitors (10). In patients with Inborn Errors of Immunity (IEI), adaptive or innate immune system defects led to gastrointestinal, respiratory, and cutaneous involvement frequently associated with dysbiosis (11, 12). Specifically, changes in gut microbiota have been described in patients with common variable immunodeficiency (CVID) due to defects in mucosal immunity and increased microbial translocation (13, 14), resulting in inflammation and immune dysregulation (13, 15). CVID is the most common IEI and is characterized by hypogammaglobulinemia, impaired antibody responses to vaccination and recurrent respiratory infections (14). About half of patients develop additional non-infectious complications such as autoimmune diseases, lymphoproliferation, and malignancies (14). In CVID, the coexistence of infection, immune dysregulation, and perturbation in microbiota-immunity interactions might lead to airway dysbiosis, contributing to the establishment of lung damage. Data on the respiratory microbiome in CVID patients are limited (16). By conventional culture methods, we previously showed the link between *H. influenzae* and *S. pneumococcus* upper respiratory tract colonization and respiratory comorbidities in CVID (16). In this single-center study, we investigated the bacterial composition of the oropharynx by molecular methods. We used the oropharynx as an easily accessible sampling site as proven to sufficiently reflect the lower airway bacterial microbiome (17–19). Our aim is to investigate whether clinical and immunological phenotypes and the extent of recurrent antibiotic use might influence oropharyngeal dysbiosis. The secondary outcome was to evaluate the link between oropharyngeal microbiota and respiratory acute infection risk in CVID.

## 2 Methods

### 2.1 Study design

**Figure 1:**
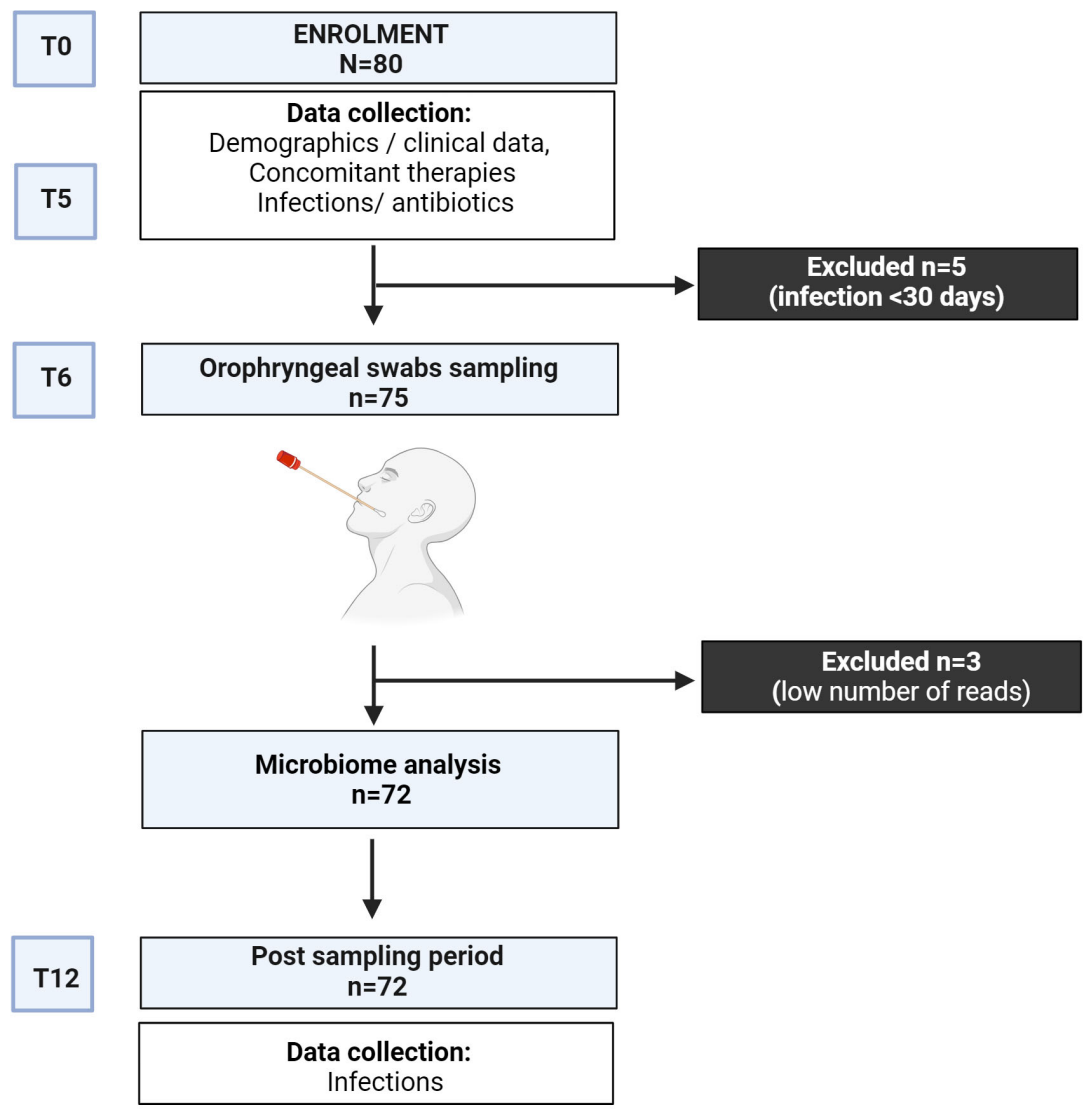
Study design.

We designed an observational longitudinal 12-month study to analyze the upper respiratory tract microbiome in adults with CVID (**Figure 1**). The study involved CVID patients aged over 18 who were regularly followed by the Referral Care Centre for Primary Immunodeficiencies at Sapienza University of Rome, Italy. Patients were diagnosed according to the ESID criteria for CVID (20). Twenty-six healthy donors (HD) recruited among administrative employees of Sapienza University were also invited to participate in the study. At baseline, we collected demographics, IgG trough levels, IgA and IgM serum levels, and peripheral immune phenotype, including frequencies of B cell and Switched Memory B cells (MBC), to group patients according to the EUROCLASS classification (21). We stratified patients as having undetectable (<0.01 g/L) or detectable (≥0.01 g/L) IgA serum levels and as having IgM serum levels above or upper 0.20 g/L (2 DS lower than the reference). We also collected CVID-related health issues, including the presence of bronchiectasis (by CT scan), chronic obstructive pulmonary disease (COPD) (22), systemic autoimmunity and autoimmune cytopenia, enteropathy, Granulomatous and Lymphocytic Interstitial Lung Diseases (GLILD) (23), and concomitant treatments to allow comparison of outcome measures. Based on the data collected, we classified patients according to the prevalent manifestations into the infective or complicated phenotype (24). After enrolment, clinicians recorded monthly respiratory infections and antibiotic courses for six months (T0-T6). Six months after enrollment (T6), oropharyngeal swabs were collected. Participants were excluded if they had experienced symptoms of acute respiratory infections or had taken antibiotics in the month before the sampling. After swab collection, respiratory acute infections were monthly recorded for additional six months (T6-T12) through interviews and clinical evaluations. During the study time, patients were allowed to continue their therapies, including immunoglobulin replacement therapy (IgRT) and antibiotic prophylaxis. The study followed the Good Clinical Practice guidelines and the Declaration of Helsinki. The Ethical Board of the Policlinico Umberto I of Rome approved this study. The patients/participants provided their written informed consent to participate in this study.

### 2.2 Sample collection, DNA extraction, and sequencing

Oropharyngeal sampling was carried out using nylon flocked swabs (Copan, Brescia, Italy) placed in milk-tryptone-glucose-glycerol (STGG) medium (25). One swab was collected from each patient, refrigerated, and transferred to the laboratory at Istituto Superiore di Sanità within four hours from collection, then stored at -80°C until further processing. Total DNA was extracted from each swab using the NucleoBond^®^ AXG DNA kit (MACHEREY-NAGEL GmbH & Co., Duren, Germany), following the manufacturer’s instructions. Briefly, samples were vortexed, and 800 μl of STGG medium was used to extract total DNA. Libraries were prepared by PCR enrichment of 16S rRNA encoding sequences using universal primers for the V3-V4 hypervariable region of the 16S rRNA gene and sequenced by MiSeq technology (Illumina, Harvard, California).

### 2.3 Serum levels of immunoglobulins

IgG, IgA and IgM serum levels were assessed by immunoturbidimetric assay according to the manufacturer’s instructions (BIO plastics, Rome, Italy).

### 2.4 B cells and T cells unbiased population identification

PBMCs were stained with the appropriate combination of fluorochrome-conjugated antibodies to identify B and T cell subsets. B cell subsets were identified based on the expression of CD19, CD27, CD24, and CD38 markers by flow-cytometry. T cells were identified based on the expression of CD3+, CD4+, CD8+ by flow-cytometry. FCS files were analyzed using the FlowJo ™ v10.8.1 software (BD, Biosciences). Prior to analysis, all samples were normalized to equal cell numbers.

### 2.5 Statistical analysis

Continuous variables were described using median and interquartile ranges (IQR), and categorical variables using frequencies and percentages. Comparisons of continuous parameters between treatment groups were calculated with a t-test if normally distributed and with a Mann-Whitney U test if not normally distributed (as tested by the Kruskal-Wallis’s test). Differences in frequencies between groups were calculated by using the χ2 exact test. Secondary analyses were performed by multiple logistic and a binomial regression model to ascertain risk factors related to alpha diversity (Chao), dysbiosis index, and acute respiratory infections recorded in the T6-T12. Odds ratios (OD) and 95% confidence intervals (CIs) were calculated. Differences were considered significant when p values**<**0.05. Analyses were carried out using SPSS software, version 18 (SPSS, Chicago, IL) and GraphPad Prism version 8.0.0 for Windows, GraphPad Software, San Diego, CA USA.

### 2.6 Metagenomic analyses

Sequence data were processed using the Quantitative Insights Into Microbial Ecology (QIIME) pipeline (v.1.9.1.) (26). Data pre-processing included sample demultiplexing and trimming of Illumina adapters and primers. Paired-end reads were first assembled into longer joined sequences, and then sequences shorter than 200 bp and greater than 1000 bp were discarded. Chimeric sequences were removed before downstream processing. Sequences were clustered *de novo* into Operational Taxonomic Units (OTUs) using UCLUST at 97% similarity (As 97% identity in rRNA genes identifies the same taxa, we clustered sequences from the same taxa together). OTUs were assigned through a comparison with the SILVA database using Mothur. Alpha and beta diversity analyses were performed at all taxonomic levels (Phylum, Class, Order, Family, and Genus) (27). Samples were first characterized in terms of alpha diversity: sample richness was explored in terms of the observed number of OTUs and Chao1 index (Chao, 1987). The t-test and the paired t-test were used to determine differences between samples. Sample-to-sample differences in microbiome composition were quantified using beta-diversity and visualized in low-dimensional space through Principal Component Analysis (PCA). PERMANOVA was used to detect global community differences in PCA. Differences in microbiome composition between CVID patients and HDs were studied by Linear discriminant analysis effect size (LEfSe) (28) to identify taxa that differed consistently between sample types. LEfSe employs the non-parametric factorial Kruskal-Wallis’s sum-rank test (a = 0.05) to identify taxa with significantly different abundances between categories, followed by Linear discriminant analysis (LDA) to estimate the effect size of each feature of the differential abundance. The differences in abundance were regarded as statistically significant when the logarithmic LDA score was >2.0. If multiple taxonomic levels with different ranks showed significance in the same taxon, the lowest-ranked taxa were regarded as responsible. All sequencing data associated with this study were uploaded to the NCBI bio project database: PRJNA747877.

### 2.7 Dysbiosis index

A CVID-specific microbial dysbiosis index was calculated according to Gevers et al. (29). We first defined CVID- and HD-taxa as the taxa enriched in CVID and HD groups, respectively. Then, we computed CVID- and HD scores by summing the relative abundance of CVID and HD taxa in each sample, respectively. Finally, the dysbiosis index was computed by subtracting the HD- and the CVID score and multiplying the result by 100, as shown in the following formula:

The equations should be inserted in editable format from the equation editor.


DI= (∑ CVIDtaxa − ∑ HDtaxa)*100

### 2.8 Microbiome classifier for CVID status

Using a linear classification model, we also investigated if taxonomic global microbial community composition alone would be sufficient to distinguish CVID patients from HD (CVID vs. HD). We used SIAMCAT version 1.8.1 (Statistical Inference of Associations between Microbial Communities And host phenoTypes, https://siamcat.embl.del/), a pipeline for analyzing microbiome data (30). SIAMCAT provides a full pipeline supporting data pre-processing, statistical association testing, and statistical modeling. Briefly, we first filtered microbial species based on their maximal abundance in any of the samples using 3% as a threshold using the filter.features function (parameters: filter.method = ‘abundance’, cutoff = 0.03). We then log-normalized microbial abundances using the normalise.features function (parameters: norm.method = “log.unit”, norm.param = list (log.n0 = 1e-06, n.p = 2, norm.margin = 1)). After splitting the data for cross-validation using the create.data.split function (parameters num.folds = 5, num.resample = 2), we trained a Random Forest classifier using the train.model function (parameters: method = “randomForest”). To perform and evaluate model predictions on the test set, we used the make.predictions and the evaluate.predictions functions. Ultimately, we generated the ROC curves and the model interpretation plot using the model.evaluation.plot and model.interpretation.plot functions. All statistical analyses were performed in R.

## 3 Results

### 3.1 Patient characteristics

Oropharyngeal samples were obtained from 80 patients. After sequencing, three samples were excluded owing to the low number of reads (below 9,000). Five patients were excluded as they had an infection treated by antibiotics in the month preceding the sampling (T5-T6). The demographic and clinical data of the 72 CVID patients included in the analysis are reported in **Table 1**. As controls, we enrolled 26 asymptomatic HD, age- and sex-matched (age, median 47.7 years, range, females 13, 50%). Frequencies of smokers were comparable among groups (**Supplementary Table 1**). All CVID patients were treated by IgRT. Forty-seven percent of participants had a diagnosis of COPD, and 50% had bronchiectasis identified at a CT scan. Twenty-eight percent of CVID patients had undetectable serum IgA (<0.01 g/L) and 28% IgM levels<0.20 g/L. Fifty-seven percent of patients were classified as having a complicated phenotype: 30.6% had one or more autoimmune diseases 16.7% GLILD, 5.6% enteritis, and 15.2% malignancy (**Table 1**). Seven patients (9.7%) received antibiotic prophylaxis with azithromycin (500 mg thrice weekly). Sixty-three antibiotic courses (range 1-5 per patient) were prescribed to 34 patients in the period T0-T5. Details on antibiotic treatments are provided in **Table 1**.

**Table 1:** Demographic, clinical, and immunological data of the CVID cohort.

	CVID n=72
Sex (female), number (%)	34 (47.2)
Age (years), median (IQR) [range]	47 (41-58) [19-77]
IgG trough serum levels (g/L), median (IQR) [range]	6.7 (6.1-7.7) [5.8-9.9]
IgA serum levels g/L, median (IQR) [range]	0.02 (0.01-0.11) [0-45]
IgA< 0.01 g/L, number (%)	20 (27.8)
IgM serum levels g/L, median (IQR) [range]	0.95 (0.20-0.24) [0-66]
IgM< 0.20 g/L, number (%)	20 (27.8)
MBC (% of lymphocytes), median (IQR)	8 (3.24-12)
Switched MBC (% of B cells) median (IQR)	1 (0-5)
Bronchiectasis, n of patients (%)	35 (50)
Complicated phenotype, n of patients (%)	41 (56.9)
COPD, n of patients (%)	
No	38 (52.8)
Yes, mild	16 (22.2)
Yes, moderate/severe	18 (25)
GLILD n of patients (%)	12 (16.7)
Concomitant treatment, n of patients (%)	
Steroids (inhalers)	17 (23.9)
Antibiotic prophylaxis	7 (9.7)
Antibiotic use (T0-T5)	34 (47)
Respiratory infections (T6-T12), n of patients (%)	31 (43)
Antibiotic course (T0-T5)	
Yes, patients (%)	28 (45.9)
Days, median (IQR)	7 (0-10)
Amoxicillin/ clavulanate, n of patients (%)	16 (25)
Trimethoprim/ sulfamethoxazole, n of patients (%)	2 (3)
Macrolides, n of patients (%)	11 (16)
Cephalosporin, n of patients (%)	6 (9)
Quinolones, n of patients (%)	10 (15)
Lincomycin, n of patients (%)	3 (4)

CVID, common variable immunodeficiency; COPD, chronic obstructive pulmonary disease; IQR interquartile range, GLILD Granulomatous and Lymphocytic Interstitial Lung Diseases; MBC, memory B cells.

### 3.2 CVID patients showed signs of oropharyngeal dysbiosis

**Figure 2:**
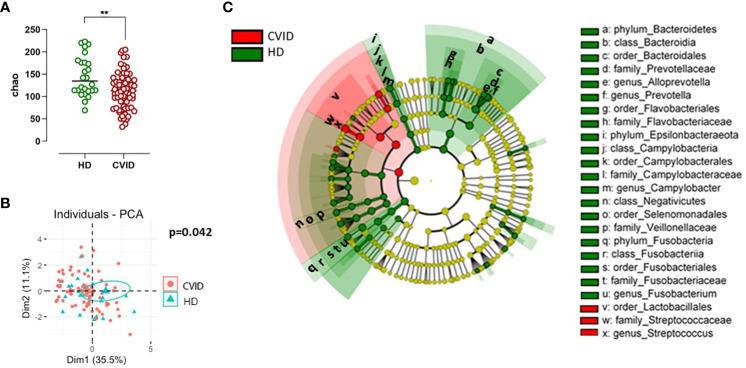
Alpha and beta diversity **(A, B)** and bacterial community composition of the oropharynx in CVID and controls **(C)**. Species richness and diversity index were estimated by Chao1 (alpha diversity) and represented in CVID vs. controls **(A)**. Bars indicate the median. Non-parametric Mann–Whitney t-test was used to evaluate statistical significance. Beta diversity by Principal Component Analysis (PCA, **B**) was calculated to capture inter-sample variation in microbial composition. Two-tailed P value significances are shown as **p< 0.01. A cladogram **(C)** illustrating the phylogenetic relationship between taxa, the central dot representing the kingdom Bacteria, the first circle representing Phylum, then the Class, Order, Family, and Genus levels. Taxa that are increased in CVID compared with controls are in red, and taxa that are reduced in CVID compared with controls are in green. Named taxa are significant according to both univariate and multivariate statistics and are marked as small letters in the cladogram referring to corresponding taxa names in the legend at the right side of the figure. The vertical lines on the left side of the legend define taxa representing different levels of the same branch. The phylogenetic tree and coloring were made using LEfSe.

**Figure 3:**
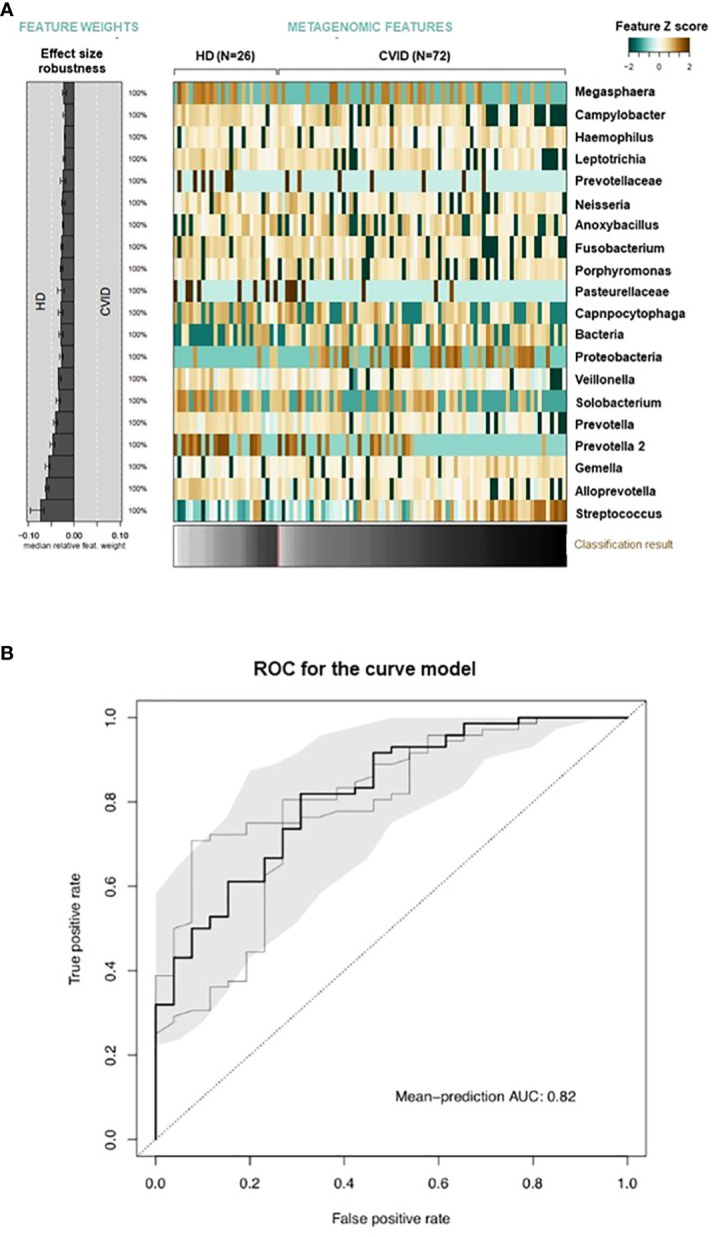
Global microbiome classifier by CVID status. Relative abundances of oropharyngeal microbial taxa associated with CVID status are displayed as a heatmap of log-abundance z-scores with the direction of association indicated to the left **(A)**. The mean contribution of each marker species to the classification is shown to the left (bars correspond to the log-odds ratio in logistic regression). Below the heatmap, the classification score of the microbial signature from cross-validation is shown as a gray scale. **(B)** The cross-validation accuracy of the microbiota classifier is depicted as a receiver–operator-characteristic (ROC) curve summarizing mean test predictions made in ten times resampled tenfold cross-validation with the area under the curve (AUC) indicated inside each plot.

According to the Chao1 index, the alpha diversity, a measure of the richness and evenness of a sample, was lower in CVID compared to HD (p=0.003, **Figure 2A**). Beta-diversity analysis, which measures differences between communities, showed a separation between HD and CVID groups (PERMANOVA p=0.042, **Figure 2B**). Microbiome profiles were also investigated in terms of taxonomic assignment and relative abundance (**Supplementary Figure 1**). We found 24 taxa at different taxonomic levels with significant differential abundance in CVID patients compared to HD, with the order *Lactobacillales*, including the family *Streptococcaceae* being more abundant in CVID, and the order *Bacteroidales*, *Flavobacteriales*, *Fusobacteriales*, *Selenomonadales*, *Campylobacterales*, including the families *Prevotellaceae*, *Fusobacteriaceae*, *Veillonellaceae*, *Campylobacteraceae*, and *Flavobacteriaceae* being more abundant in HD (**Figure 2C**; **Supplementary Table 3**). To quantify the strength of the association between CVID status (CVID *vs*. HD) and global oropharyngeal microbiome observed by metagenomic analysis, we also used a classification concept based on LASSO regression (30). Cross-validation accuracy was high for CVID status (mean AUC=82%), confirming a different global oropharyngeal microbiome composition in CVID and HD (**Figure 3**).

### 3.3 Perturbation in oropharyngeal microbiota in CVID is related to immunological defects, clinical manifestations, and antibiotic treatments

**Figure 4:**
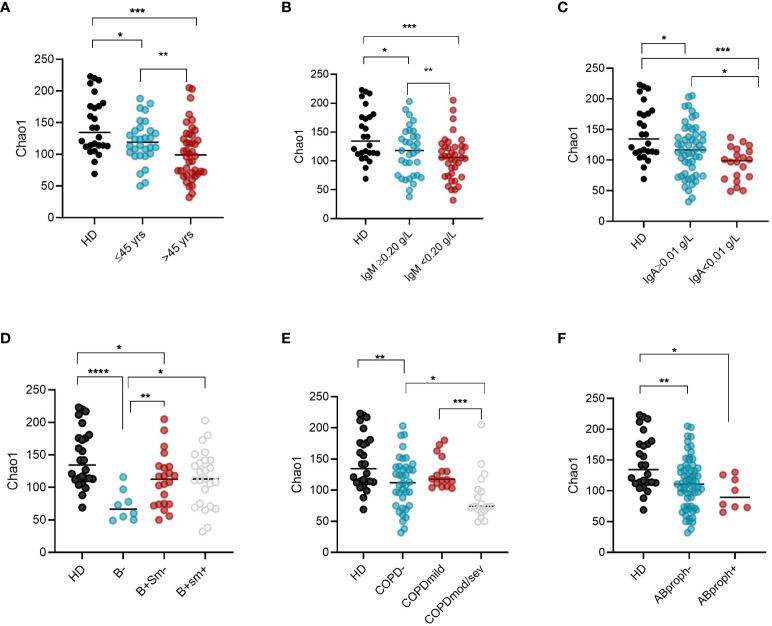
Intra-CVID differences in alpha diversity in oropharyngeal microbiome. Chao1 (alpha diversity) was compared in CVID patients grouped according to their age **(A)**, IgM **(B)** and IgA serum levels **(C)**, EUROCLASS groupin0g **(D)**, COPD status **(E)**, and whether to take or not antibiotic prophylaxis **(F)**. Bars indicate the median. Non-parametric Mann–Whitney t-test was used to evaluate statistical significance. Two-tailed P value significances are shown as * p<0.05, **p< 0.01, ***p< 0.001. ****p<0.0001. HD, healthy donors; yrs, years; B-, B cells<1% of lymphocytes; B+Sm-, B cells>1% and Switched Memory B cells >=2% of lymphocytes; B+Sm+, B cells>1% and Switched Memory B cells >2% of lymphocytes; COPD, chronic obstructive pulmonary diseases; mod/sev, moderate-severe; AB, prophylaxis antibiotic prophylaxis.

**Figure 5:**
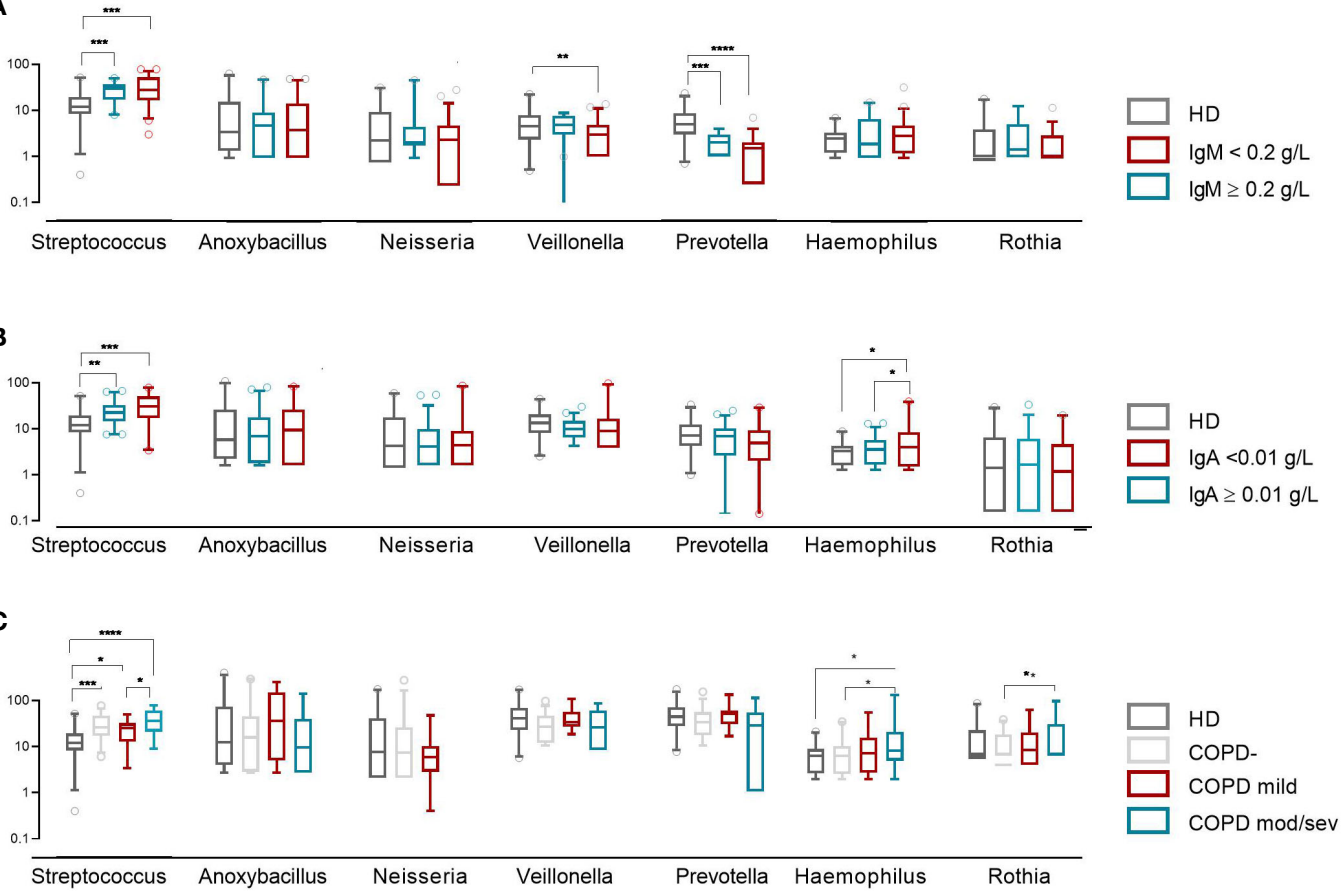
Bacterial community composition of the oropharynx in CVID patients and controls. Patients were grouped by IgM and IgA serum levels **(A, B)** and COPD status **(C)**. The first six most frequently identified genera in CVID patients are shown. The horizontal line inside the box represents the median. The whiskers represent the 10 and 90 percentiles. The non-parametric Mann–Whitney test was used to evaluate statistical significance. Two-tailed P value significances are shown as *p<0.05, **p< 0.01, ***p< 0.001, ****p<0.0001.

Intra-CVID differences in species richness and diversity (Chao1) were analyzed by univariate analysis, revealing a decreased microbial alpha diversity associated with age ≥45 years, low IgM serum levels (<0.20 g/dL), undetectable IgA serum levels (<0.1 g/dL), low B cells (≤1% of lymphocytes), and severe/moderate COPD status (**Figures 4A–E**; **Supplementary Table 2**). A multiple logistic regression model using a stepwise selection confirmed that the Chao1 index was related to IgM defect, age, and COPD moderate/severe status (**Table 2**). A reduction trend in Chao1 was observed in those treated with antibiotic prophylaxis, even if not statistically significant (**Figure 4F**). Differently, bronchiectasis, days of antibiotic treatments (T0-T5), GLILD coexistence, having a CVID complicated phenotype, and the concomitant treatment with steroid inhalers were not associated with a reduction in alpha diversity in this cohort (**Supplementary Table 2**). At the study time, all participants were receiving immunoglobulin replacement treatment (median IgG TL 6.7 g/L, **Table 1**). No contribution of IgG TL to the Chao1 index was observed in this cohort (**Supplementary Table 2**). Beta-diversity analyses revealed a significant separation between CVID lacking IgA and CVID with detectable IgA serum levels (PERMANOVA p = 0.014, **Supplementary Figure 2**). We further analyzed the impact of the identified variables on the bacterial community composition. Compared to those with IgM ≥0.20 g/L, CVID patients with IgM**<** 0.20 g/L had lower levels of *Prevotella* and *Veillonella*, two genera usually related to a healthy status (**Figure 5A**). Furthermore, patients with undetectable IgA serum levels had a higher relative abundance of the genera *Haemophilus* and *Streptococcus* compared to those with serum IgA levels ≥0.01 g/L (**Figure 5B**). A similar pattern was observed in CVID patients with moderate/severe COPD, who showed higher relative abundance for *Streptococcus*, *Haemophilus*, and *Rothia* genera compared with patients with mild or without COPD (**Figure 5C**). Compared to controls, patients >45 years had an even higher abundance of *Streptococcus* genera and lower levels of *Prevotella* and *Veillonella* than younger patients (**Supplementary Figure 3**). No differences in bacterial community composition were identified among the seven patients treated with azithromycin prophylaxis. We further analyzed the effect of antibiotic therapy to treat acute respiratory infection, only identifying a reduced relative abundance of *Prevotella* in those treated with one or more courses of quinolones (p=0,008).

**Table 2:** Variables associated with alpha diversity in CVID patients.

	P value	OR	95% CI	
≥45 years	0,020	-22,250	-40,890	-3,611
COPD mod/severe	0,030	-22,284	-42,372	-2,197
IgM ≥ 0.2 g/L	0,039	0,596	0,030	1,162

CI, confidence intervals; COPD, chronic obstructive pulmonary disease; OR, odds ratio. A linear logistic regression model using a stepwise selection procedure was calculated. Odds ratios (OR) and 95% confidence intervals (CI) of multivariate models are reported.

### 3.4 CVID-specific dysbiosis index

To explore the possible impact of disease-related variables on the overall severity of dysbiosis, we calculated a CVID-specific dysbiosis index. We used the relative abundance of the taxa differentiating CVID patients and controls to calculate the index according to the method as previously described (12, 29). The dysbiosis index was higher in CVID patients than in HD. It was inversely associated with the alpha diversity (R 0.6 p<0.0001, **Supplementary Figure 4**), confirming that the abundance of selected taxa primarily captures the dysbiosis in CVID patients. In a linear regression model, the dysbiosis index was directly related to be treated by prophylaxis with macrolides (OR 31.9, p=0.009, 95%CI 8,6 to 55.3), and negatively related to being female (OR -21.4, p=0.006, 95%CI from -36.2 to -6.7) (**Table 3**). As only few patients were treated with antibiotic prophylaxis, we also tested the model after removing its contribution and we further identified the moderate/severe COPD status as having a direct relation with dysbiosis index (OR 18.7, p=0.036, 95%CI from 1.3 to 36.1) (**Supplementary Table 4**).

**Table 3:** Linear logistic model using a stepwise selection procedure to assess the impact of CVID-related characteristics collected during the study on dysbiosis index.

	OR	P value
Final Model		
Sex	-21.4	.006
Antibiotic prophylaxis	31.9	.009
Excluded variables:		
Age >=45 years	0.1	0.681
Smokers (yes)	-0.2	0.126
IgA< 0.01 g/L	0.1	0.518
IgM >0.20 g/L	-0.1	0.523
IgG TL g/L	-0.1	0.550
SwMBC<=2% of B cells	0.1	0.686
Complicated phenotype	-0.2	0.298
COPD moderate/severe	-0.1	0.515
Bronchiectasis	-0.1	0.934
Systemic autoimmunity	-0.1	0.953
Antibiotic use (T0-T5)	-0.2	0.219
Days of antibiotic therapy	-0.2	0.129
Amoxicillin/clavulanate (days)	-0.2	0.215
Trimethoprim/sulfamethoxazole (days)	-0.1	0.660
Macrolides (days)	0.1	0.590
Cephalosporin (days)	-0.3	0.074
Quinolones (days)	-0.2	0.183
Lincomycin (days)	0.2	0.252
Corticosteroids treatment by inhaler (T0-T6)	0.0	0.816

COPD, chronic obstructive pulmonary diseases; OR, odds ratio; CI, confidence intervals; SwMBC, switched memory B cells.

### 3.5 Respiratory acute infections correlated with *Haemophilus* and *Anoxybacillus*

Next, we investigated whether the observed oropharyngeal microbiota differences in CVID patients were associated with infections in the T6-T12 period. In binomial regression model including all CVID participants, *Haemophilus* (p = 0.029, OR 5.52, 95%CI 0.606- 10.427, **Supplementary Table 5**) positively correlated with having a respiratory acute infection within 60 days from sampling, whereas *Anoxybacillus* (p=0.026, OR 1.47, CI95% 0.18-2.74) positively correlated with having a respiratory acute infection within six months (**Supplementary Table 6**).

## 4 Discussion

Over the last two decades, 16S rRNA amplicon sequencing has brought significant understanding into the architecture of the human microbiome and its role in health and illness (7). Several reports on respiratory diseases have indicated that the imbalance in microbiome composition can affect disease progression and severity, raising inflammation and acute lung damage accompanied by symptom exacerbations (31). Due to their immune defect, CVID patients are at high risk for developing recurrent infections, particularly by encapsulated bacteria (32), evolving towards lung damage (33) with a negative impact on the quality of life and survival (34).

In this study, we analyzed the composition of the oropharynx microbiome in a cohort of CVID adults and the link between CVID-associated conditions, recurrent antibiotic use, and altered respiratory microbiome composition. Our data revealed a strong association between the oropharyngeal microbiome niche composition and the CVID status. Compared to healthy controls, CVID patients exhibited a reduced diversity in the respiratory microbiome and the expansion of potentially pathogenic bacteria. This distinct microbiome signature was also confirmed by a machine-learned-based comparative metagenomics tool, a classification model recently validated in microbiome analysis (30).

Beta diversity analyses identified that CVID samples were gathered separately from controls, with CVID having undetectable IgA serum levels clustering separately. At taxonomic levels, in CVID, we observed the relative expansion of the genus Streptococcus and the decreasing abundance of the families *Prevotellaceae, Fusobacteriaceae, Veillonellaceae, Campylobacteraceae*, and *Flavobacteriaceae*, the most abundant phyla of healthy lung microbiomes (6). A similar depletion is observed in respiratory conditions such as pneumonia, COPD, and in smokers (32–37), together with the overgrowth of *P.aeruginosa*, *S.pneumoniae, S.aureus, H. Influenza*, and *Burkholderia cepacia* complex (11), leading to increased inflammation and lung injury (29). In our CVID cohort, respiratory acute infections have been positively related to the expansion of *Haemophilus* and *Anoxybacillus*, which has been previously associated with lung function worsening in Idiopathic Pulmonary Fibrosis (35) and exacerbations in asthma and COPD (38, 39). This data was coherent with the high *H.influenzae* and *S.pneumoniae* mucosal carriage previously identified in CVID by culture method (40). In healthy airways, low biomass of species belonging to the *Streptococcus* and *Haemophilus* genera are commonly colonizing pathobionts (41). Chronic inflammation favors the growth of selected species (42). These can disseminate and cause infections (43), establishing a vicious cycle between oral dysbiosis and respiratory diseases (2, 44).

When we stratified CVID patients according to their immunological characteristics, we found that the lack of IgA, low IgM levels, older age, and reduced circulating B cells affected the alpha diversity reduction. While IgA defect is included among the CVID diagnostic criteria, some patients are entirely IgA deficient, whereas others have a residual IgA production (14). Previously, our group showed that low IgA levels together with reduced frequencies of peripheral and mucosal memory B cells represent a risk for a worse prognosis, infections, and lung damage in CVID (45–47). Under healthy conditions, secretory IgA shapes the microbiome and neutralizes toxins and viruses without activating the complement cascade. Moreover, IgA blocks the colonization of pathogenic bacteria by binding receptors on the fimbriae, clearing unwanted particles, and promoting the sampling of antigens (8–10). Here, we recorded a reduced alpha diversity in the subset of patients with low IgA, partially confirming the data from Barbers et al. (16). In addition, we recorded that the lack of IgA was associated with the expansion of the *Haemophilus* and *Streptococcus* genera. The role of IgM in microbiome homeostasis remains less understood (12). Here, we recorded a more accentuated microbiological pathological signature in patients with a severe depletion of IgM, with lower levels of *Prevotella* and *Veillonella*, two genera usually related to healthy status. In this context, the impossibility of replacing IgA and IgM at the mucosal level should be considered (48). This emphasizes the need for possible additional therapeutic interventions, such as aerosolized IgA/IgM, to prevent bacterial dysbiosis in the respiratory tract in CVID.

We also observed a lower diversity and dominance of the genera *Haemophilus, Streptococcus* and *Rothia* in patients with co-existing severe/moderate COPD, in line with data from non-CVID cohorts with severe COPD (31). In COPD, *Streptococcus* expansion and its associated metabolites have been previously related to worsening lung function and respiratory exacerbations (49). In addition, *Haemophilus* dominance has been associated with airway neutrophil inflammation and disease severity (49, 50). These data suggest that perturbation in the respiratory microbiome in CVID patients with COPD might contribute to lung disease progression and morbidity.

Given the frequent use of antibiotics in patients with CVID, the study also aimed to investigate the extent to which recurrent antibiotic use might influence oropharyngeal dysbiosis. In our CVID cohort we identified the antibiotic prophylaxis as one of the main factor influencing dysbiosis. This data was in line with the observation of reduced α-diversity and lower relative abundance of respiratory pathogens such as *Pseudomonas aeruginosa*, *Moraxella catarrhalis* and members of family *Enterobacteriaceae* in the airway microbiome of COPD patients receiving prophylactic antibiotics, together with a high frequency of resistance to macrolide and tetracycline (51). In contrast, there was no difference in microbial diversity and composition between CVID patients treated with antibiotics as needed and those who were not treated. This is probably due to the high complexity of CVID, where the clinical and immunological factors discussed above lead to changes in the microbiota even in patients who have not been treated with antibiotics.

In the gut, the main immunological trait associated with gut dysbiosis was systemic dysregulation and low IgA (13, 15), leading to the expansion of *Bacilli* and *Gammaproteobacteria* and increased microbial translocation (13). Our data suggests that oropharyngeal dysbiosis is influenced mainly by disruption in microbiota-immunity balance and by local inflammation resulting in COPD. Similarly, oropharyngeal dysbiosis in CVID has been previously associated with radiographic lung damage severity (16).

The study’s main limitation is the choice of the oropharynx as a sampling site. However, in healthy conditions and inflammatory respiratory diseases (17–20), the lung microbiota community is similar to the oropharyngeal microbiome. Moreover, the role of viruses as causative agents of acute respiratory infections was not explored since the 16S rRNA sequencing method is incapable of detecting viral DNA. A further limitation is the lack of longitudinal sampling making a single time point evaluation interesting but not enough to fully address the microbiome composition since it might change over time (52).

**Figure 6:**
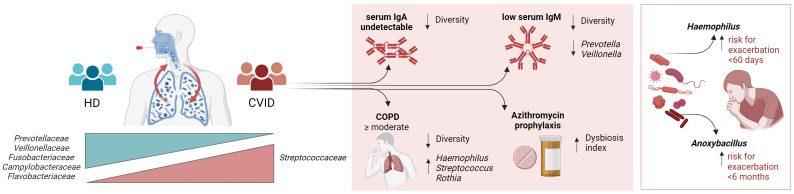
Summary of the main results.

In conclusion, our results demonstrate that the oropharyngeal niche of CVID patients exhibits a distinct microbiome structure characterized by reduced diversity, enrichment of potentially pathogenic bacteria, and decreased protective species. We have also found that respiratory acute infections are linked to the expansion of distinct oropharyngeal bacterial taxa. Additionally, we have observed that the subset of CVID patients with COPD or undetectable IgA displays a microbiome ecosystem that is enriched with *Streptococcus* and *Haemophilus*, which may act as a source of infection and inflammation (**Figure 6**). Our findings suggest that manipulating the respiratory microbiota through pharmacological modification or replacing the immune defect may shape the microbiota composition and reduce inflammation and damage progression. Moreover, given the immunological interactions in the gut-lung axis (53), treatment with immunobiotics might also gain attention, considering their potential to confer protection against infections by modulating innate and adaptive antimicrobial immunity (54). Similarly, as intestinal probiotics have been shown to reduce the number and duration of upper respiratory tract infections (55, 56), their use can potentially contrast respiratory infections.

## Data availability statement

Sequence data were submitted to GenBank at NCBI under the Bio Project and are available at https://www.ncbi.nlm.nih.gov/bioproject/PRJNA747877.

## Ethics statement

The studies involving humans were approved by The ethics committee/institutional review board of Sapienza University of Rome, Italy. The studies were conducted in accordance with the local legislation and institutional requirements. The participants provided their written informed consent to participate in this study.

## Author contributions

FP: Data curation, Formal analysis, Investigation, Methodology, Project administration, Writing – original draft, Writing – review & editing. MG: Conceptualization, Methodology, Writing – original draft, Writing – review & editing, Formal analysis. TP: Formal analysis, Methodology, Writing – original draft, Writing – review & editing. RC: Methodology, Writing – original draft, Writing – review & editing. CM: Writing – review & editing, Investigation. AV: Writing – review & editing, Investigation. ES: Writing – review & editing, Investigation. MC: Writing – review & editing, Methodology, Resources. AP: Writing – review & editing, Funding acquisition, Methodology, Resources. IQ: Writing – review & editing, Conceptualization, Funding acquisition, Methodology, Resources, Writing – original draft.
