# Supplementary material for: Oropharyngeal microbial ecosystem perturbations influence the risk for acute respiratory infections in common variable immunodeficiency
Source: Front Immunol. 2024 May 30;15:1371118. doi: 10.3389/fimmu.2024.1371118 (PMC11169596; doi:10.3389/fimmu.2024.1371118)

Supplementary materials.

1. **Supplementary Tables**

**Supplementary Table 1.** Overview of the characteristics for CVID and controls

|  | **CVID (n=72)** | **HD (n=26)** | **P value** |
| --- | --- | --- | --- |
| **Age in years** mean (SD),  [range] | 48,6 (12,7)  [19-77] | 47,7 (11,9)  [27-61] | 0.759 |
| **Sex, female** n (%) | 34 (47.2) | 13 (50) | 0.059 |
| **Smokers** n (%) | 4 (5.0) | 2 (7,7) | 0.086 |

Abbreviations: CVID, common variable immunodeficiency; HD healthy donors

**Supplementary Table 2.** Contribution of demographic, clinical, and immunological data on alpha diversity (Chao and Shannon).

|  | **Chao1 index**  **mean (SD)** | **R2** | **P value** |
| --- | --- | --- | --- |
| Sex |  |  |  |
| Females | 116.3 (39.1) | – | 0.199 |
| Males | 104.2 (40.2) | – |  |
| Age |  |  |  |
| >45 years | 114.3 (45.4) | – | 0.016 |
| <=45 years | 137.4 (38.7) | – |  |
| Smokers |  |  |  |
| Yes | 151.8 (41.9) | – | 0.085 |
| No | 118.7 (45.2) | – |  |
| Immunological data |  |  |  |
| IgG Trough level |  |  |  |
| >= 0.6 g/L | 115.2 (39.2) | – | 0.078 |
| <0.6 g/L | 96.9 (39.8) | – |  |
| IgA serum levels |  |  |  |
| > = 0.01 g/L | 116.1 (42.4) | – | 0.011 |
| < 0.01 g/L | 93.9 (27.1) | – |  |
| IgM serum levels |  |  |  |
| > 0.20 g/L | 126,6 (35.4) | – | 0.018 |
| <= 0.20 g/L | 102.6 (39.8) | – |  |
| Switched MBC |  |  |  |
| <=2% of B cells | 112.3 (42.4) | – | 0.827 |
| > 2% of B cells | 109.7 (41.0) | – |  |
| Clinical manifestations |  |  |  |
| Bronchiectasis |  |  |  |
| Yes | 113.1 (36.5) | – | 0.457 |
| No | 106.4 (41.8) | – |  |
| Complicated phenotype |  |  |  |
| Yes | 103.3 (37.4) | – | 0.107 |
| No | 118.6 (42.0) | – |  |
| COPD |  |  |  |
| No/Yes mild | 89.2 (38.8) | – | 0.010 |
| Yes, moderate/severe | 116.8 (38.1) | – |  |
| Granulomatous lung diseases (GLILD) |  |  |  |
| Yes | 118.5 (40.51) | – | 0.354 |
| No | 107.7 (39.4) | – |  |
| Concomitant treatment (T0-T5) |  |  |  |
| Steroids (inhalers) |  |  |  |
| Yes | 97.3 (31.3) | – | 0.151 |
| No | 113.3 (41.9) | – |  |
| Antibiotic prophylaxis |  |  |  |
| Yes | 90.7 (24.2) | – | 0.182 |
| No | 112.0 (40.8) | – |  |
| Antibiotic use, days |  | 0.001 | 0.843 |

Abbreviations, CVID common variable immunodeficiency, COPD chronic obstructive pulmonary disease, IQR interquartile range.

**Supplementary Table 3**. Bacterial taxa with different abundance in CVID and controls.

| Taxon | Increased in | LDA Effect size | LEfSe  *P* value | *t* test  *P* value |
| --- | --- | --- | --- | --- |
| Bacteria.Bacteroidetes | HD | 4.63 | 8.7x10^-5^ | 8.5 x10^-4^ |
| Bacteria.Bacteroidetes.Bacteroidia | HD | 4.63 | 8.7x10^-5^ | 8.5 x10^-4^ |
| Bacteria.Bacteroidetes.Bacteroidia.Bacteroidales | HD | 4.58 | 1.8 x10^-5^ | 0.0010 |
| Bacteria.Bacteroidetes.Bacteroidia.Bacteroidales.Prevotellaceae | HD | 4.50 | 5.1 x10^-4^ | 0.0011 |
| Bacteria.Bacteroidetes.Bacteroidia.Bacteroidales.Prevotellaceae.Alloprevotella | HD | 4.14 | 1.9 x10^-4^ | 4.1 x10^-5^ |
| Bacteria.Bacteroidetes.Bacteroidia.Bacteroidales.Prevotellaceae.Prevotella | HD | 3.68 | 2.0 x10^-4^ | 0.0273 |
| Bacteria.Bacteroidetes.Bacteroidia.Flavobacteriales | HD | 3.69 | 0.0046 | 0.0178 |
| Bacteria.Bacteroidetes.Bacteroidia.Flavobacteriales.Flavobacteriaceae | HD | 3.58 | 0.0109 | 0.0326 |
| Bacteria.Fusobacteria | HD | 4.26 | 0.0183 | 0.0062 |
| Bacteria.Fusobacteria.Fusobacteriia | HD | 4.26 | 0.0183 | 0.0062 |
| Bacteria.Fusobacteria.Fusobacteriia.Fusobacteriales | HD | 4.26 | 0.0183 | 0.0062 |
| Bacteria.Fusobacteria.Fusobacteriia.Fusobacteriales.Fusobacteriaceae | HD | 3.99 | 0.0355 | 0.0042 |
| Bacteria.Fusobacteria.Fusobacteriia.Fusobacteriales.Fusobacteriaceae.Fusobacterium | HD | 3.99 | 0.0355 | 0.0042 |
| Bacteria.Firmicutes.Negativicutes | HD | 4.20 | 0.0402 | 0.0035 |
| Bacteria.Firmicutes.Negativicutes.Selenomonadales | HD | 4.20 | 0.0402 | 0.0035 |
| Bacteria.Firmicutes.Negativicutes.Selenomonadales.Veillonellaceae | HD | 4.20 | 0.0402 | 0.0035 |
| Bacteria.Epsilonbacteraeota | HD | 3.54 | 0.0219 | 0.0284 |
| Bacteria.Epsilonbacteraeota.Campylobacteria | HD | 3.54 | 0.0219 | 0.0284 |
| Bacteria.Epsilonbacteraeota.Campylobacteria.Campylobacterales | HD | 3.54 | 0.0219 | 0.0250 |
| Bacteria.Epsilonbacteraeota.Campylobacteria.Campylobacterales.Campylobacteraceae | HD | 3.54 | 0.0231 | 0.0293 |
| Bacteria.Epsilonbacteraeota.Campylobacteria.Campylobacterales.Campylobacteraceae.Campylobacter | HD | 3.54 | 0.0231 | 0.0293 |
| Bacteria.Firmicutes.Bacilli.Lactobacillales | CVID | 4.97 | 2.8x10^-6^ | 0.0042 |
| Bacteria.Firmicutes.Bacilli.Lactobacillales.Streptococcaceae | CVID | 4.94 | 5.5x10^-6^ | 0.0050 |
| Bacteria.Firmicutes.Bacilli.Lactobacillales.Streptococcaceae.Streptococcus | CVID | 4.95 | 9.3x10^-5^ | 0.0045 |

**Supplementary Table 4.** Linear logistic model using a stepwise selection procedure to assess the impact of CVID-related characteristics collected during the study on dysbiosis index. The variable antibiotic prophylaxis has been here removed.

|  | **OR** | **P value** |
| --- | --- | --- |
| **Final Model** |  |  |
| Sex (female) | -18.5 | 0.019 |
| COPD (moderate/severe) | 18.7 | 0.036 |
| **Excluded variables:** |  |  |
| Age ≥45 years | 0.0 | 0.829 |
| Smokers | -0.2 | 0.179 |
| IgA < 0.01 g/L | 0.1 | 0.388 |
| IgM ≥0.20 g/L | -0.1 | 0.332 |
| IgG TL g/L | 0.0 | 0.695 |
| SwMBC ≤ 2% of B cells | 0.0 | 0.664 |
| Complicated phenotype | -0.1 | 0.654 |
| Bronchiectasis | -0.1 | 0.855. |
| Systemic autoimmunity | 0.0 | 0.795 |
| Antibiotic use (T0-T5) | -0.1 | 0.348 |
| Days of antibiotic therapy | -0.2 | 0.167 |
| Amoxicillin/clavulanate (days) | -0.2 | 0.134 |
| Trimethoprim/sulfamethoxazole (days) | -0.1 | 0.735 |
| Macrolides (days) | 0.10 | 0.511 |
| Cephalosporin (days) | -0.2 | 0.112 |
| Quinolones (days) | -0.1 | 0.528 |
| Lincomycin (days) | 0.2 | 0.272 |
| Corticosteroids treatment by inhaler (T0-T6) | 0.1 | 0.652 |

Abbreviations: COPD chronic obstructive pulmonary diseases, OR odds ratio, CI confidence intervals, SwMBC switched memory B cells.

**Supplementary Table 5.** Binomial logistic model using a stepwise selection procedure to assess the risk for respiratory tract exacerbation within 60 days (T6-T8). Odds ratios (OR) and 95% confidence intervals (CI) of multivariate models are reported.

| **Final Model** | **OR** | **P value** |
| --- | --- | --- |
| Haemophilus | 20.4 | 0.017 |
| **Excluded variables:** | **OR** | **P value** |
| Anoxybacillus | -0.1 | 0.213 |
| Rothia | 0.1 | 0.435 |
| Alloprevotella | 0.1 | 0.877 |
| Prevotella | 0.1 | 0.279 |
| Gemella | -0.1 | 0.400 |
| Streptococcus | -0.1 | 0.685 |
| Veilonella | 0.2 | 0.060 |
| Neisseria | -0.1 | 0.890 |

**Supplementary Table 6.** Binomial logistic model using a stepwise selection procedure to assess the risk for respiratory tract exacerbation within 6 months (T6-T12). Odds ratios (OR) and 95% confidence intervals (CI) of multivariate models are reported.

|  | **OR** | **P value** |
| --- | --- | --- |
| **Final Model** |  |  |
| Anoxybacillus | 10.5 | 0.026 |
| **Excluded variables:** | **OR** | **P value** |
| Rothia | 0.1 | 0.612 |
| Alloprevotella | 0.1 | 0.554 |
| Prevotella | 0.0 | 0.949 |
| Gemella | 0.1 | 0.647 |
| Streptococcus | 0.0 | 0.775 |
| Veilonella | 0.0 | 0.950 |
| Neisseria | 0.1 | 0.352 |
| Haemophilus | 0.2 | 0.335 |

**2. Supplementary Figures**

**Supplementary Figure 1. Colour-coded bar plots show bacterial taxa's average distribution at phylum0. class0. order0. families0. and genus levels in HD and CVID.** Only taxa with a mean relative abundance ≥1% are shown. Taxa were sorted by decreasing mean relative abundances in the HD group.


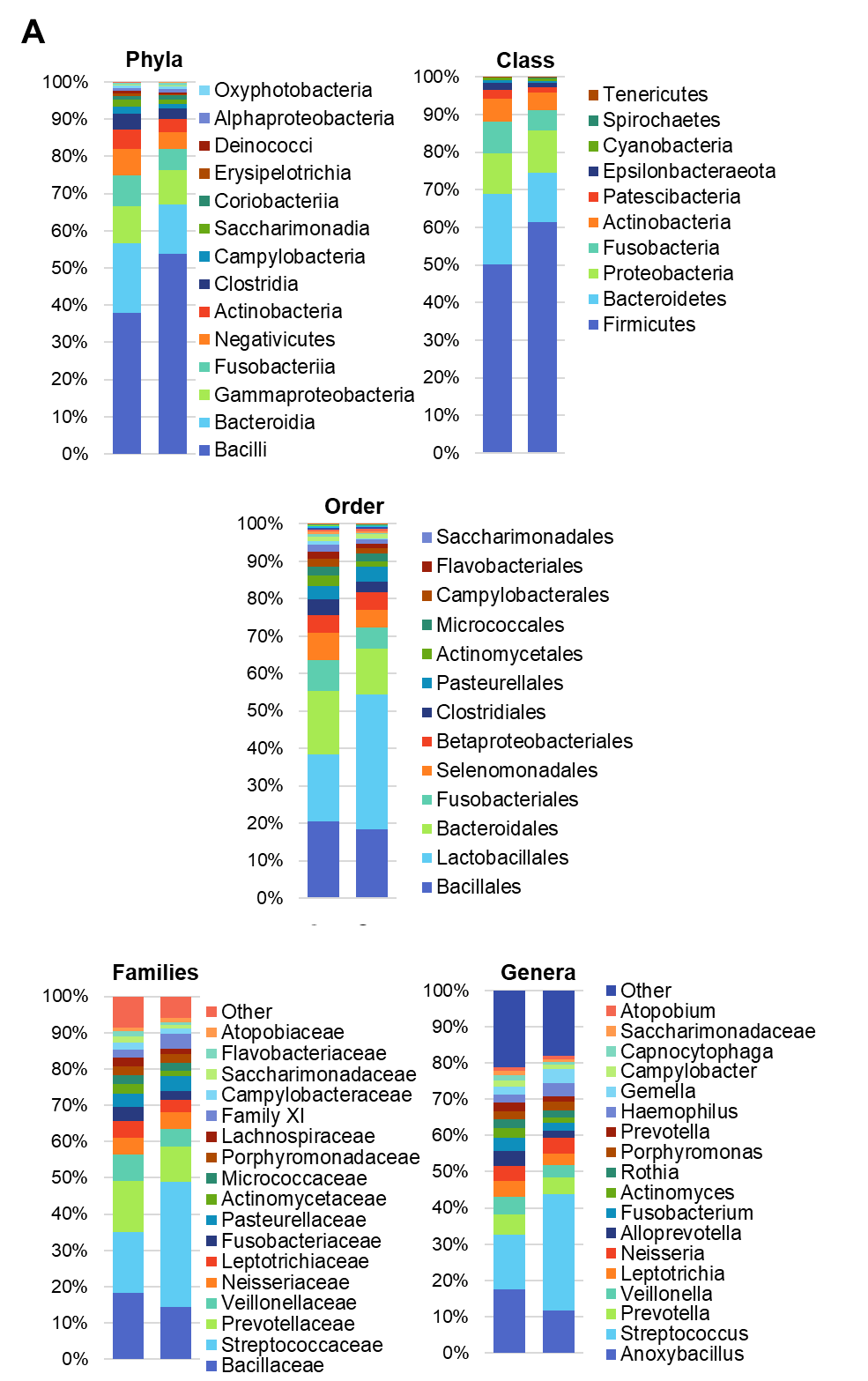


**Supplementary Figure 2. Beta diversity by Principal Component Analysis (PCA) between controls and CVID with/without IgA undetectable serum levels is shown**

**
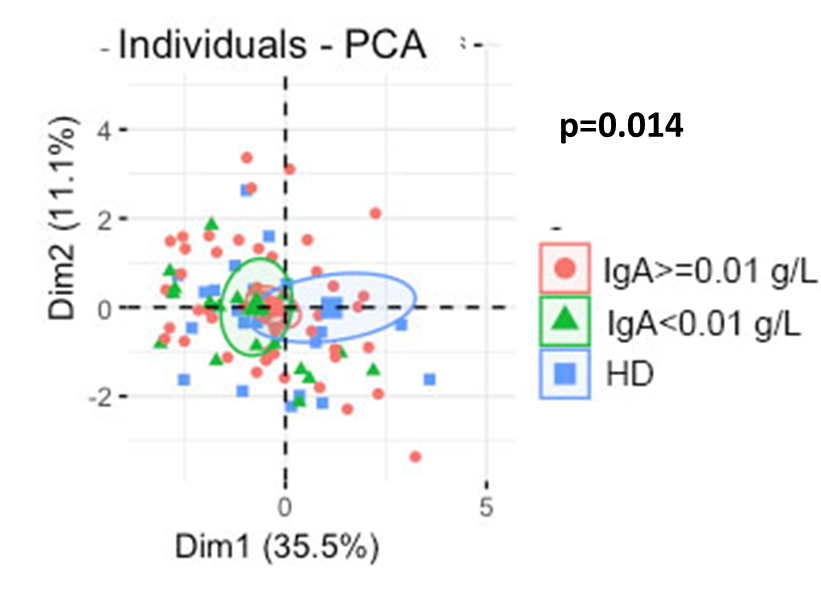
**

**Supplementary Figure 3**. **Bacterial community composition of the oropharynx in CVID patients grouped by their age and controls.** The first six most frequently identified genera in CVID patients are shown. Lines represent the median. The whiskers represent the 10 and 90 percentiles. The non-parametric Mann–Whitney test was used to evaluate statistical significance. Two-tailed P value significances are shown as *p<0.050. **p < 0.010. ***p < 0.0010. ****p<0.0001. Abbreviations: HD healthy donors


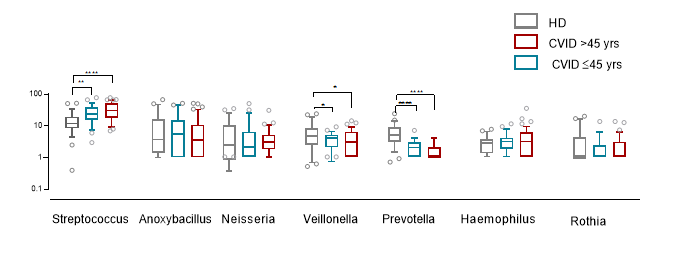


**Supplementary Figure 4. CVID-dysbiosis index.** The relative abundance of the 11 main taxa in CVID patients and controls (CVID-dysbiosis index). The dysbiosis index was higher in CVID patients than in HD (OR 30.00. 95%CI 19.8 to 40.20. panel A) and inversely related to the alpha diversity (OR -1.10. 95%CI -1.3 to -0.80. panel B). Levels of significance: ****p<0.0001


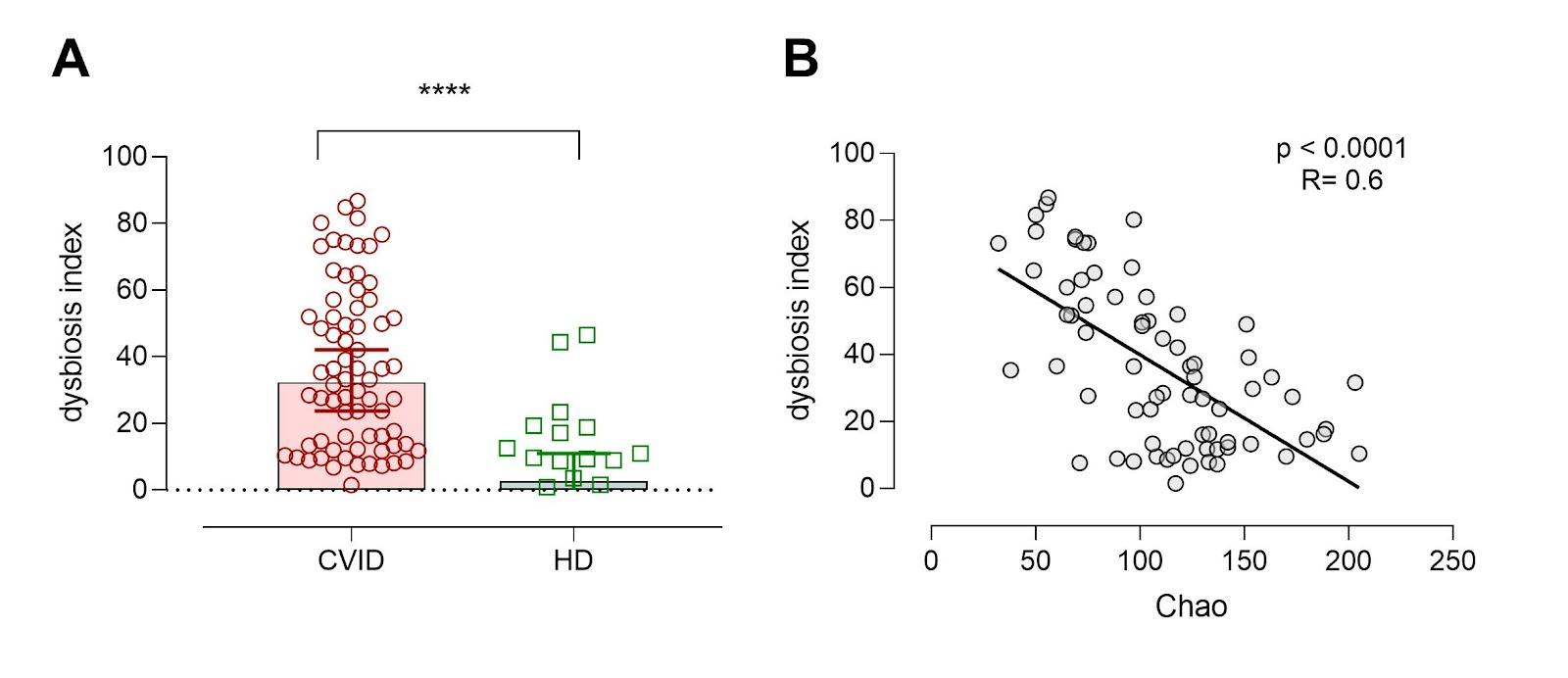

Supplement: Supplementary file 1 [file DataSheet_1.docx]
